# Supplementary material for: Causal Relationship between Aging and Anorexia Nervosa: A White-Matter-Microstructure-Mediated Mendelian Randomization Analysis
Source: Biomedicines. 2024 Aug 16;12(8):1874. doi: 10.3390/biomedicines12081874 (PMC11351342; doi:10.3390/biomedicines12081874)
Supplement: Supplementary file 1 [file biomedicines-12-01874-s001.zip › Supplementary Figure/Figure S8/Leaveoneout plot of MR analysis for TL to ubm-b-2007.pdf]

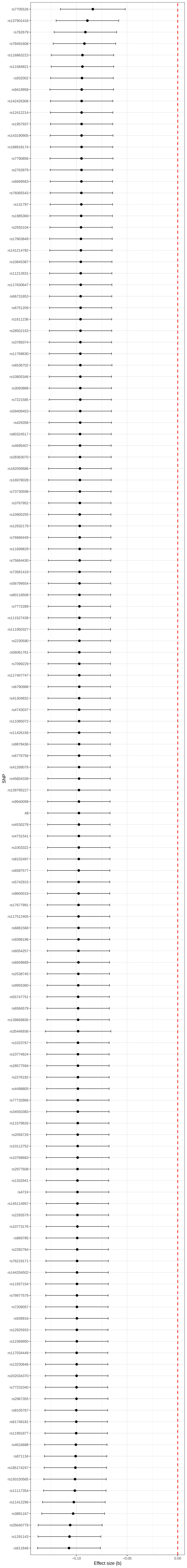

rs7705526
rs137901416
rs762679
rs78491606
rs116863223
rs11584821
rs932002
rs9419958
rs142426306
rs12412214
rs1957937
rs143190905
rs188918174
rs7790856
rs2763979
rs6669563
rs76065543
rs131797
rs1985369
rs2555104
rs17803849
rs141214782
rs10845387
rs11212631
rs117630647
rs66731853
rs6751209
rs1611236
rs28502153
rs3785074
rs11769630
rs6536702
rs10805346
rs3093888
rs7221585
rs59409453
rs429358
rs80324517
rs4695407
rs28363070
rs182059586
rs16978028
rs73730598
rs3767952
rs10905255
rs12932179
rs76666449
rs11699829
rs75664430
rs73581419
rs56799554
rs80116508
rs7772289
rs111527438
rs111950327
rs2230590
rs56061761
rs7099229
rs117407747
rs6790988
rs41304832
rs4743037
rs11085072
rs11426156
rs9878436
rs6776756
rs41269079
rs45604339
rs139795227
rs9940099
All
rs4530278
rs4731541
rs1003322
rs8102497
rs6587577
rs5742915
rs9600019
rs17677991
rs117512405
rs6881568
rs9398196
rs6054257
rs6659669
rs2538745
rs9955360
rs55747751
rs6584579
rs139669835
rs35446936
rs1023767
rs10774624
rs28577594
rs2276182
rs4498805
rs77732866
rs34550383
rs11579626
rs2056726
rs10112752
rs10768683
rs2977608
rs1332941
rs4724
rs145114957
rs2293579
rs10773176
rs869785
rs2282764
rs76219171
rs144204502
rs11557154
rs79977579
rs7209057
rs939916
rs12925933
rs12369950
rs117034449
rs13230646
rs202034370
rs77231040
rs2967355
rs8105767
rs61748181
rs11991877
rs4616688
rs871134
rs185174247
rs150150565
rs11117354
rs11412296
rs3891167
rs35640778
rs1291143
rs611646
SNP
-0.10
-0.05
0.00
Effect size (b)
